# [μ-Bis(diphenyl­arsino)methane-1:2κ^2^ *As*:*As*′]nona­carbonyl-1κ^3^ *C*,2κ^3^ *C*,3κ^3^ *C*-[diphen­yl(phenyl­sulfanylmeth­yl)phosphine-3κ*P*]-*triangulo*-triruthenium(0) chloro­form hemisolvate

**DOI:** 10.1107/S1600536810001212

**Published:** 2010-01-30

**Authors:** Omar bin Shawkataly, Imthyaz Ahmed Khan, Chin Sing Yeap, Hoong-Kun Fun

**Affiliations:** a Chemical Sciences Programme, School of Distance Education, Universiti Sains Malaysia, 11800 USM, Penang, Malaysia; b X-ray Crystallography Unit, School of Physics, Universiti Sains Malaysia, 11800 USM, Penang, Malaysia

## Abstract

The asymmetric unit of the title *triangulo*-triruthenium cluster, [Ru_3_(C_25_H_22_As_2_)(C_19_H_17_PS)(CO)_9_]·0.5CHCl_3_, contains of one mol­ecule of the *triangulo*-triruthenium complex and half a mol­ecule of the disordered (two positions of equal weight) chloro­form solvent. The bis­(diphenyl­arsino)methane ligand bridges an Ru—Ru bond and the monodentate phosphine ligand bonds to the third Ru atom. Both the arsine and phosphine ligands are equatorial with respect to the Ru_3_ triangle. In addition, each Ru atom carries one equatorial and two axial terminal carbonyl ligands. The benzene ring of phenyl­thio­methyl is disordered over two positions with refined site occupancies of 0.788 (11) and 0.212 (11). In the crystal packing, mol­ecules are linked into chains along *b* axis by inter­molecular C—H⋯O hydrogen bonds. Weak inter­molecular C—H⋯π inter­actions further stabilize the crystal structure.

## Related literature

For general background to *triangulo*-triruthenium derivatives, see: Bruce *et al.* (1985 ▶, 1988*a* ▶,*b* ▶). For related structures, see: Shawkataly *et al.* (1998 ▶, 2004 ▶, 2009 ▶). For the synthesis of μ-bis­(diphenyl­arsino)methane­deca­carbonyl­triruthenium(0), see: Bruce *et al.* (1983 ▶). For the synthesis of diphen­yl(phenyl­thio)methyl­phosphine, see: Sanger (1983 ▶). For the stability of the temperature controller used for the data collection, see: Cosier & Glazer (1986 ▶).
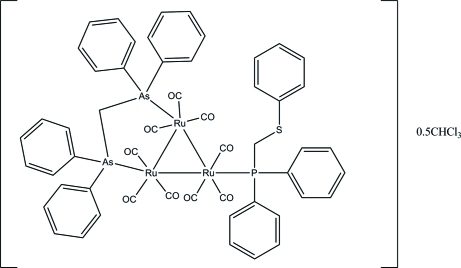


## Experimental

### 

#### Crystal data

[Ru_3_(C_25_H_22_As_2_)(C_19_H_17_PS)(CO)_9_]·0.5CHCl_3_
*M* *_r_* = 1395.61
Monoclinic, 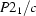

*a* = 23.0129 (4) Å
*b* = 11.6027 (2) Å
*c* = 20.5019 (4) Å
β = 92.876 (1)°
*V* = 5467.35 (17) Å^3^
*Z* = 4
Mo *K*α radiation
μ = 2.21 mm^−1^
*T* = 100 K
0.51 × 0.16 × 0.04 mm

#### Data collection

Bruker SMART APEXII CCD area-detector diffractometer
Absorption correction: multi-scan (*SADABS*; Bruker, 2005 ▶) *T*_min_ = 0.399, *T*_max_ = 0.923
70928 measured reflections
16008 independent reflections
11323 reflections with *I* > 2σ(*I*)
*R*_int_ = 0.065

#### Refinement

*R*[*F* ^2^ > 2σ(*F* ^2^)] = 0.059
*wR*(*F* ^2^) = 0.158
*S* = 1.03
16008 reflections
678 parameters
162 restraints
H-atom parameters constrained
Δρ_max_ = 1.78 e Å^−3^
Δρ_min_ = −3.30 e Å^−3^

### 

Data collection: *APEX2* (Bruker, 2005 ▶); cell refinement: *SAINT* (Bruker, 2005 ▶); data reduction: *SAINT*; program(s) used to solve structure: *SHELXTL* (Sheldrick, 2008 ▶); program(s) used to refine structure: *SHELXTL*; molecular graphics: *SHELXTL*; software used to prepare material for publication: *SHELXTL* and *PLATON* (Spek, 2009 ▶).

## Supplementary Material

Crystal structure: contains datablocks global, I. DOI: 10.1107/S1600536810001212/tk2610sup1.cif

Structure factors: contains datablocks I. DOI: 10.1107/S1600536810001212/tk2610Isup2.hkl

Additional supplementary materials:  crystallographic information; 3D view; checkCIF report

## Figures and Tables

**Table 1:** Hydrogen-bond geometry (Å, °) *Cg*1, *Cg*2 and *Cg*3 are the centroids of the C39*A*–C44*A*, C14–C19 and C32–C37 benzene rings, respectively.

*D*—H⋯*A*	*D*—H	H⋯*A*	*D*⋯*A*	*D*—H⋯*A*
C18—H18*A*⋯O1^i^	0.93	2.53	3.457 (7)	175
C29—H29*A*⋯O3^ii^	0.93	2.45	3.331 (9)	158
C3—H3*A*⋯*Cg*1^iii^	0.93	2.82	3.654 (9)	151
C23—H23*A*⋯*Cg*2^iv^	0.93	2.87	3.691 (7)	147
C44*A*—H44*A*⋯*Cg*3	0.93	2.76	3.526 (12)	141

Symmetry codes: (i) 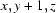; (ii) 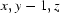; (iii) 
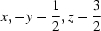
; (iv) 
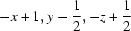
.

**Fig. 1.**
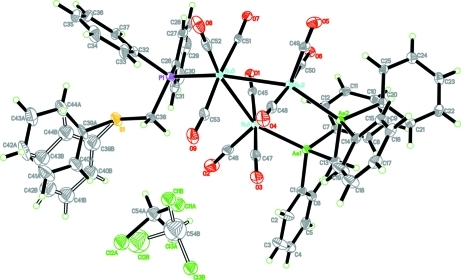
The molecular structure of the title compound with 30% probability ellipsoids for non-H atoms. All disordered components are shown.

**Fig. 2.**
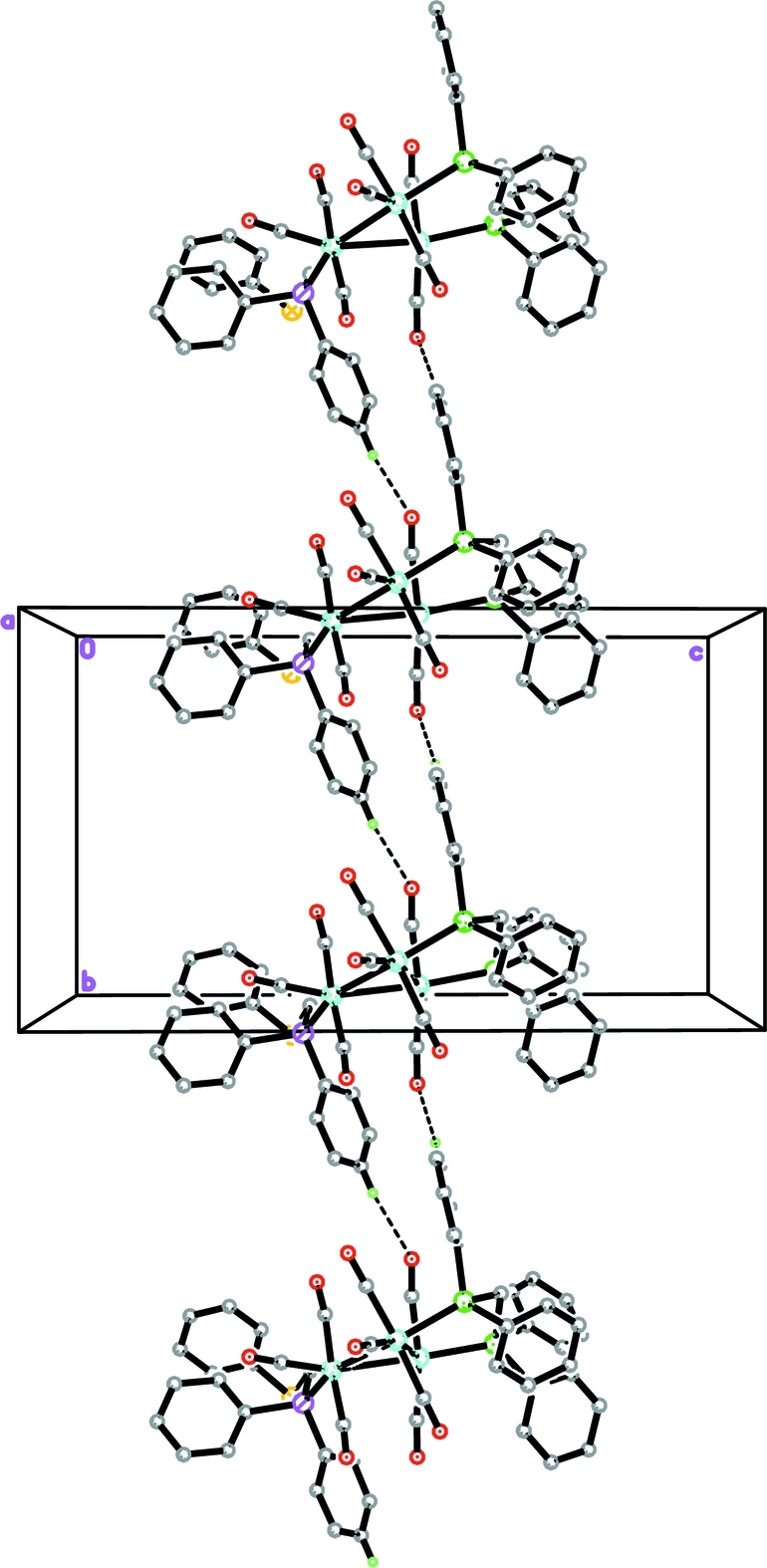
The crystal packing of the title compound, viewed down the a axis, showing the molecules linked into a chain along b axis by C–H···O contacts (dashed lines); the remaining H atoms and the solvent molecules have been deleted for reasons of clarity. Only major disordered component is shown.

## supplementary crystallographic information

### Comment

*Triangulo*-triruthenium clusters are known for their interesting structural variations and related catalytic activity. A large number of substituted derivatives, Ru_3_(CO)_12-n_*L*_n_ (*L*= group 15 ligand) have been reported (Bruce *et al.*, 1985, 1988*a*,b. As part of our study on the substitution of transition metal-carbonyl clusters with mixed-ligand complexes, we have published several structures of *triangulo*-triruthenium-carbonyl clusters containing mixed P/As and P/Sb ligands (Shawkataly *et al.*, 1998, 2004, 2009). Herein we report the synthesis and structure of title compound.

The asymmetric unit consists of one molecule of the *triangulo*-triruthenium complex and half molecule of disordered chloroform solvent (Fig. 1). The bond lengths and angles of title compound are comparable to those found in a related structure (Shawkataly *et al.*, 2009). The bis(diphenylarsino)methane ligand bridges the Ru1—Ru2 bond and the monodentate phosphine ligand bonds to the Ru3 atom. Both the phosphine and arsine ligands are equatorial with respect to the Ru_3_ triangle. Additionally, each Ru atom carries one equatorial and two axial terminal carbonyl ligands. The phosphine-substituted benzene rings (C26–C31/C39–C44 and C32–C37/C39–C44) make dihedral angles of 51.1 (5) and 57.8 (5)° with the benzene ring of phenylthiomethyl residue for the major component whereas these values are 42 (2) and 67 (2)° for the minor component. The dihedral angles between the two benzene rings (C1–C6/C7–C12 and C14–C19/C20–C25) are 78.7 (3) and 76.8 (3)° for the two diphenylarsino groups respectively.

In the crystal packing (Fig. 2), the molecules are linked into chains along *b* axis by intermolecular C18—H18A···O1 and C29—H29A···O3 contacts. Weak intermolecular C—H···π interactions further stabilize the crystal structure (Table 1).

### Experimental

All manipulations were performed under a dry, oxygen-free dinitrogen atmosphere using standard Schlenk techniques. All solvents were dried over sodium and distilled from sodium benzophenone ketyl under nitrogen. Diphenyl(phenylthiomethyl)phosphine (Sanger, 1983) was used as received and µ-bis(diphenylarsino)methane-decacarbonyl-triruthenium(0) was prepared by a reported procedure (Bruce *et al.*, 1983). The title compound was obtained by refluxing equimolar quantities of Ru_3_(CO)_10_(µ-Ph_2_AsCH_2_AsPh_2_) (105.5 mg, 0.1 mmol) and diphenyl(phenylthiomethyl)phosphine (30.84 mg, 0.1 mmol) in hexane under a nitrogen atmosphere. Crystals were grown by slow solvent / solvent diffusion of C_6_H_14_ into CHCl_3_.

### Refinement

All hydrogen atoms were positioned geometrically and refined using a riding model with C—H = 0.93–0.97 Å and *U*_iso_(H) = 1.2 *U*_eq_(C). The C39–C44 ring and the chloroform molecule are disordered over two positions. The benzene ring has refined site occupancies of 0.788 (11) and 0.212 (11) for C39–C44 ring whereas site occupancies of the chloroform molecule are fixed to 0.25 for both components at final refinement. The same U_ij_ parameters were used for the atom pairs Cl1B/Cl3B, Cl1B/Cl1A and C41B/C39B. The disordered atoms of the ring were subjected to rigid bond and similarity restraints and the minor component is refined isotropically. The chloroform atoms were only subjected to rigid bond restraint and both components are refined isotropically. The maximum and minimum residual electron density peaks of 1.78 and -3.30 e Å^-3^, respectively, were located 0.77 Å and 0.07 Å from the Cl2B and Cl3B atoms, respectively.

### Crystal data

**Table:**

[Ru_3_(C_25_H_22_As_2_)(C_19_H_17_PS)(CO)_9_]·0.5CHCl_3_	*F*(000) = 2748
*M**_r_* = 1395.61	*D*_x_ = 1.695 Mg m^−^^3^
Monoclinic, *P*2_1_/*c*	Mo *K*α radiation, λ = 0.71073 Å
Hall symbol: -P 2ybc	Cell parameters from 9929 reflections
*a* = 23.0129 (4) Å	θ = 2.2–28.3°
*b* = 11.6027 (2) Å	µ = 2.21 mm^−^^1^
*c* = 20.5019 (4) Å	*T* = 100 K
β = 92.876 (1)°	Plate, brown
*V* = 5467.35 (17) Å^3^	0.51 × 0.16 × 0.04 mm
*Z* = 4	

### Data collection

**Table:**

Bruker SMART APEXII CCD area-detector diffractometer	16008 independent reflections
Radiation source: fine-focus sealed tube	11323 reflections with *I* > 2σ(*I*)
graphite	*R*_int_ = 0.065
φ and ω scans	θ_max_ = 30.1°, θ_min_ = 2.0°
Absorption correction: multi-scan (*SADABS*; Bruker, 2005)	*h* = −32→31
*T*_min_ = 0.399, *T*_max_ = 0.923	*k* = −16→16
70928 measured reflections	*l* = −26→28

### Refinement

**Table:**

Refinement on *F*^2^	Primary atom site location: structure-invariant direct methods
Least-squares matrix: full	Secondary atom site location: difference Fourier map
*R*[*F*^2^ > 2σ(*F*^2^)] = 0.059	Hydrogen site location: inferred from neighbouring sites
*wR*(*F*^2^) = 0.158	H-atom parameters constrained
*S* = 1.03	*w* = 1/[σ^2^(*F*_o_^2^) + (0.0739*P*)^2^ + 20.3511*P*] where *P* = (*F*_o_^2^ + 2*F*_c_^2^)/3
16008 reflections	(Δ/σ)_max_ = 0.001
678 parameters	Δρ_max_ = 1.78 e Å^−^^3^
162 restraints	Δρ_min_ = −3.30 e Å^−^^3^

### Special details

**Table:**

Experimental. The crystal was placed in the cold stream of an Oxford Cyrosystems Cobra open-flow nitrogen cryostat (Cosier & Glazer, 1986) operating at 100.0 (1) K.
Geometry. All e.s.d.'s (except the e.s.d. in the dihedral angle between two l.s. planes) are estimated using the full covariance matrix. The cell e.s.d.'s are taken into account individually in the estimation of e.s.d.'s in distances, angles and torsion angles; correlations between e.s.d.'s in cell parameters are only used when they are defined by crystal symmetry. An approximate (isotropic) treatment of cell e.s.d.'s is used for estimating e.s.d.'s involving l.s. planes.
Refinement. Refinement of *F*^2^ against ALL reflections. The weighted *R*-factor *wR* and goodness of fit *S* are based on *F*^2^, conventional *R*-factors *R* are based on *F*, with *F* set to zero for negative *F*^2^. The threshold expression of *F*^2^ > σ(*F*^2^) is used only for calculating *R*-factors(gt) *etc*. and is not relevant to the choice of reflections for refinement. *R*-factors based on *F*^2^ are statistically about twice as large as those based on *F*, and *R*- factors based on ALL data will be even larger.

### Fractional atomic coordinates and isotropic or equivalent isotropic displacement parameters (Å^2^)

**Table:**

	*x*	*y*	*z*	*U*_iso_*/*U*_eq_	Occ. (<1)
Ru1	0.752259 (18)	0.04613 (3)	0.46155 (2)	0.02268 (10)	
Ru2	0.638973 (17)	0.11916 (3)	0.49541 (2)	0.02088 (10)	
Ru3	0.716761 (18)	0.02270 (3)	0.59312 (2)	0.02310 (10)	
As1	0.72787 (2)	0.08678 (4)	0.34618 (3)	0.02365 (12)	
As2	0.61767 (2)	0.22498 (4)	0.39438 (3)	0.02128 (12)	
P1	0.79472 (6)	−0.08541 (11)	0.63827 (7)	0.0223 (3)	
S1	0.92881 (7)	−0.10436 (16)	0.65861 (10)	0.0492 (5)	
O1	0.73103 (17)	−0.2152 (3)	0.46385 (19)	0.0273 (8)	
O2	0.8804 (2)	−0.0041 (5)	0.4513 (3)	0.0661 (17)	
O3	0.7739 (2)	0.3063 (4)	0.4722 (2)	0.0426 (11)	
O4	0.6689 (2)	0.3441 (4)	0.5684 (2)	0.0457 (12)	
O5	0.5215 (2)	0.1306 (4)	0.5558 (3)	0.0456 (12)	
O6	0.60819 (17)	−0.1138 (3)	0.4309 (2)	0.0296 (9)	
O7	0.63677 (18)	−0.1879 (4)	0.5702 (2)	0.0348 (10)	
O8	0.6529 (2)	0.0746 (5)	0.7156 (3)	0.0559 (15)	
O9	0.7890 (2)	0.2424 (4)	0.6171 (3)	0.0548 (14)	
C1	0.7947 (2)	0.1213 (5)	0.2947 (3)	0.0298 (12)	
C2	0.8323 (3)	0.2062 (7)	0.3161 (4)	0.054 (2)	
H2A	0.8244	0.2490	0.3529	0.064*	
C3	0.8826 (3)	0.2289 (7)	0.2828 (4)	0.059 (2)	
H3A	0.9075	0.2877	0.2973	0.070*	
C4	0.8955 (3)	0.1660 (7)	0.2296 (4)	0.0465 (17)	
H4A	0.9292	0.1805	0.2078	0.056*	
C5	0.8576 (3)	0.0800 (7)	0.2084 (3)	0.0455 (17)	
H5A	0.8660	0.0363	0.1720	0.055*	
C6	0.8074 (3)	0.0579 (6)	0.2403 (3)	0.0356 (13)	
H6A	0.7821	0.0002	0.2252	0.043*	
C7	0.6829 (2)	−0.0168 (4)	0.2886 (3)	0.0246 (11)	
C8	0.6530 (3)	0.0243 (5)	0.2326 (3)	0.0324 (13)	
H8A	0.6544	0.1022	0.2221	0.039*	
C9	0.6212 (3)	−0.0516 (6)	0.1925 (3)	0.0410 (15)	
H9A	0.6022	−0.0247	0.1544	0.049*	
C10	0.6175 (3)	−0.1666 (6)	0.2088 (3)	0.0356 (14)	
H10A	0.5947	−0.2162	0.1827	0.043*	
C11	0.6474 (3)	−0.2079 (5)	0.2636 (3)	0.0353 (13)	
H11A	0.6457	−0.2859	0.2739	0.042*	
C12	0.6804 (3)	−0.1332 (5)	0.3035 (3)	0.0290 (12)	
H12A	0.7009	−0.1615	0.3403	0.035*	
C13	0.6838 (2)	0.2317 (4)	0.3380 (3)	0.0251 (11)	
H13A	0.7088	0.2961	0.3507	0.030*	
H13B	0.6700	0.2429	0.2929	0.030*	
C14	0.5972 (2)	0.3862 (4)	0.4061 (3)	0.0255 (11)	
C15	0.5393 (2)	0.4151 (5)	0.4107 (3)	0.0273 (11)	
H15A	0.5105	0.3596	0.4036	0.033*	
C16	0.5243 (3)	0.5280 (5)	0.4262 (3)	0.0306 (12)	
H16A	0.4854	0.5475	0.4296	0.037*	
C17	0.5663 (3)	0.6101 (5)	0.4363 (3)	0.0360 (14)	
H17A	0.5559	0.6850	0.4470	0.043*	
C18	0.6231 (3)	0.5827 (5)	0.4310 (4)	0.0505 (19)	
H18A	0.6515	0.6391	0.4372	0.061*	
C19	0.6390 (3)	0.4695 (5)	0.4161 (4)	0.0465 (18)	
H19A	0.6781	0.4508	0.4130	0.056*	
C20	0.5559 (2)	0.1743 (4)	0.3329 (3)	0.0223 (10)	
C21	0.5464 (2)	0.2288 (5)	0.2732 (3)	0.0281 (11)	
H21A	0.5684	0.2928	0.2630	0.034*	
C22	0.5039 (3)	0.1879 (5)	0.2288 (3)	0.0330 (13)	
H22A	0.4973	0.2252	0.1890	0.040*	
C23	0.4717 (2)	0.0927 (5)	0.2432 (3)	0.0303 (12)	
H23A	0.4443	0.0640	0.2125	0.036*	
C24	0.4799 (3)	0.0392 (5)	0.3038 (3)	0.0323 (13)	
H24A	0.4574	−0.0241	0.3141	0.039*	
C25	0.5218 (2)	0.0806 (4)	0.3486 (3)	0.0258 (11)	
H25A	0.5272	0.0456	0.3892	0.031*	
C26	0.8014 (2)	−0.2287 (5)	0.6040 (3)	0.0276 (11)	
C27	0.7589 (3)	−0.3107 (5)	0.6167 (3)	0.0340 (13)	
H27A	0.7289	−0.2923	0.6435	0.041*	
C28	0.7618 (4)	−0.4194 (6)	0.5891 (4)	0.051 (2)	
H28A	0.7334	−0.4738	0.5975	0.062*	
C29	0.8059 (5)	−0.4477 (6)	0.5497 (4)	0.064 (3)	
H29A	0.8075	−0.5211	0.5317	0.077*	
C30	0.8473 (4)	−0.3685 (6)	0.5370 (4)	0.053 (2)	
H30A	0.8774	−0.3889	0.5106	0.064*	
C31	0.8458 (3)	−0.2566 (5)	0.5629 (3)	0.0373 (14)	
H31A	0.8737	−0.2023	0.5529	0.045*	
C32	0.7982 (2)	−0.1102 (6)	0.7268 (3)	0.0352 (14)	
C33	0.7886 (3)	−0.0200 (7)	0.7672 (3)	0.0451 (17)	
H33A	0.7783	0.0511	0.7492	0.054*	
C34	0.7940 (3)	−0.0314 (9)	0.8353 (4)	0.062 (2)	
H34A	0.7879	0.0317	0.8620	0.075*	
C35	0.8082 (4)	−0.1367 (9)	0.8621 (4)	0.066 (3)	
H35A	0.8113	−0.1455	0.9073	0.079*	
C36	0.8178 (4)	−0.2289 (8)	0.8223 (4)	0.061 (2)	
H36A	0.8271	−0.3002	0.8408	0.074*	
C37	0.8138 (3)	−0.2173 (6)	0.7544 (3)	0.0407 (16)	
H37A	0.8214	−0.2798	0.7277	0.049*	
C38	0.8670 (2)	−0.0170 (5)	0.6294 (3)	0.0339 (13)	
H38A	0.8713	0.0005	0.5837	0.041*	
H38B	0.8680	0.0553	0.6531	0.041*	
C39A	0.9630 (4)	−0.0187 (8)	0.7212 (6)	0.049 (2)	0.788 (11)
C40A	1.0030 (4)	0.0658 (9)	0.7095 (7)	0.061 (3)	0.788 (11)
H40A	1.0119	0.0829	0.6668	0.073*	0.788 (11)
C41A	1.0300 (6)	0.1252 (11)	0.7607 (9)	0.070 (3)	0.788 (11)
H41A	1.0563	0.1833	0.7519	0.084*	0.788 (11)
C42A	1.0195 (5)	0.1017 (10)	0.8222 (8)	0.070 (3)	0.788 (11)
H42A	1.0395	0.1407	0.8560	0.084*	0.788 (11)
C43A	0.9786 (5)	0.0188 (10)	0.8361 (6)	0.070 (3)	0.788 (11)
H43A	0.9701	0.0032	0.8791	0.084*	0.788 (11)
C44A	0.9501 (5)	−0.0410 (8)	0.7846 (6)	0.058 (3)	0.788 (11)
H44A	0.9223	−0.0963	0.7934	0.069*	0.788 (11)
C39B	0.971 (3)	−0.014 (6)	0.690 (3)	0.075 (8)*	0.212 (11)
C40B	1.0143 (19)	0.029 (4)	0.660 (2)	0.066 (10)*	0.212 (11)
H40B	1.0186	0.0090	0.6171	0.079*	0.212 (11)
C41B	1.056 (2)	0.106 (4)	0.691 (3)	0.075 (8)*	0.212 (11)
H41B	1.0874	0.1295	0.6676	0.090*	0.212 (11)
C42B	1.051 (3)	0.140 (6)	0.741 (3)	0.077 (11)*	0.212 (11)
H42B	1.0719	0.2052	0.7539	0.092*	0.212 (11)
C43B	1.018 (3)	0.092 (5)	0.782 (3)	0.069 (9)*	0.212 (11)
H43B	1.0225	0.1078	0.8265	0.083*	0.212 (11)
C44B	0.973 (2)	0.013 (4)	0.756 (3)	0.065 (8)*	0.212 (11)
H44B	0.9467	−0.0194	0.7833	0.078*	0.212 (11)
C45	0.7361 (2)	−0.1166 (5)	0.4646 (3)	0.0236 (10)	
C46	0.8316 (3)	0.0137 (5)	0.4562 (3)	0.0374 (14)	
C47	0.7636 (3)	0.2091 (5)	0.4722 (3)	0.0326 (13)	
C48	0.6615 (3)	0.2591 (5)	0.5411 (3)	0.0322 (13)	
C49	0.5659 (2)	0.1267 (4)	0.5333 (3)	0.0279 (12)	
C50	0.6223 (2)	−0.0288 (4)	0.4552 (3)	0.0250 (11)	
C51	0.6668 (2)	−0.1098 (5)	0.5754 (2)	0.0256 (11)	
C52	0.6771 (3)	0.0533 (6)	0.6688 (3)	0.0363 (14)	
C53	0.7619 (3)	0.1615 (5)	0.6044 (3)	0.0356 (14)	
Cl1A	0.9091 (4)	0.2695 (8)	0.5442 (4)	0.0537 (13)*	0.25
Cl2A	1.0368 (4)	0.2562 (8)	0.5737 (5)	0.066 (2)*	0.25
Cl3A	0.9950 (5)	0.1735 (10)	0.4648 (5)	0.088 (3)*	0.25
C54A	0.9738 (6)	0.190 (3)	0.5415 (10)	0.061 (8)*	0.25
H54A	0.9689	0.1142	0.5619	0.073*	0.25
Cl1B	0.9122 (4)	0.2591 (8)	0.5652 (4)	0.0537 (13)*	0.25
Cl2B	1.0312 (7)	0.2160 (18)	0.5333 (11)	0.161 (7)*	0.25
Cl3B	0.9727 (3)	0.3836 (7)	0.4626 (4)	0.0537 (7)*	0.25
C54B	0.9609 (10)	0.252 (3)	0.5020 (16)	0.15 (2)*	0.25
H54B	0.9474	0.1944	0.4699	0.176*	0.25

### Atomic displacement parameters (Å^2^)

**Table:**

	*U*^11^	*U*^22^	*U*^33^	*U*^12^	*U*^13^	*U*^23^
Ru1	0.0241 (2)	0.01585 (18)	0.0278 (2)	−0.00002 (14)	−0.00183 (16)	−0.00130 (16)
Ru2	0.02351 (19)	0.01277 (17)	0.0260 (2)	−0.00059 (14)	−0.00255 (16)	−0.00028 (15)
Ru3	0.0256 (2)	0.01866 (19)	0.0243 (2)	−0.00049 (15)	−0.00546 (16)	−0.00374 (16)
As1	0.0248 (3)	0.0184 (2)	0.0277 (3)	−0.00137 (19)	0.0010 (2)	0.0014 (2)
As2	0.0232 (2)	0.0125 (2)	0.0278 (3)	−0.00199 (18)	−0.0019 (2)	0.0021 (2)
P1	0.0255 (6)	0.0175 (6)	0.0234 (7)	−0.0031 (5)	−0.0036 (5)	−0.0004 (5)
S1	0.0326 (8)	0.0445 (10)	0.0689 (13)	0.0019 (7)	−0.0148 (8)	0.0074 (9)
O1	0.037 (2)	0.0171 (17)	0.027 (2)	0.0008 (15)	−0.0026 (16)	−0.0019 (15)
O2	0.034 (3)	0.074 (4)	0.091 (5)	0.016 (3)	0.018 (3)	0.028 (3)
O3	0.048 (3)	0.020 (2)	0.059 (3)	−0.0072 (18)	−0.009 (2)	−0.002 (2)
O4	0.054 (3)	0.024 (2)	0.057 (3)	−0.0008 (19)	−0.013 (2)	−0.011 (2)
O5	0.038 (2)	0.039 (3)	0.061 (3)	0.011 (2)	0.021 (2)	0.013 (2)
O6	0.033 (2)	0.0163 (17)	0.039 (2)	−0.0027 (14)	−0.0032 (17)	−0.0027 (16)
O7	0.041 (2)	0.033 (2)	0.030 (2)	−0.0149 (18)	−0.0051 (18)	0.0044 (18)
O8	0.038 (3)	0.085 (4)	0.045 (3)	0.005 (3)	−0.002 (2)	−0.036 (3)
O9	0.065 (3)	0.027 (2)	0.069 (4)	−0.014 (2)	−0.033 (3)	−0.002 (2)
C1	0.024 (3)	0.029 (3)	0.037 (3)	0.000 (2)	0.004 (2)	0.004 (2)
C2	0.046 (4)	0.051 (4)	0.067 (5)	−0.022 (3)	0.025 (4)	−0.023 (4)
C3	0.038 (4)	0.061 (5)	0.078 (6)	−0.014 (3)	0.014 (4)	−0.009 (4)
C4	0.029 (3)	0.061 (5)	0.050 (4)	0.003 (3)	0.007 (3)	0.010 (4)
C5	0.037 (3)	0.070 (5)	0.030 (3)	0.007 (3)	0.003 (3)	0.007 (3)
C6	0.030 (3)	0.044 (4)	0.033 (3)	−0.001 (2)	0.000 (2)	0.003 (3)
C7	0.026 (2)	0.022 (2)	0.025 (3)	−0.0020 (19)	−0.001 (2)	−0.004 (2)
C8	0.033 (3)	0.031 (3)	0.033 (3)	0.002 (2)	−0.002 (2)	0.002 (2)
C9	0.038 (3)	0.053 (4)	0.031 (3)	0.000 (3)	−0.009 (3)	−0.001 (3)
C10	0.033 (3)	0.044 (4)	0.030 (3)	−0.013 (3)	0.004 (2)	−0.006 (3)
C11	0.045 (3)	0.032 (3)	0.029 (3)	−0.013 (3)	0.004 (3)	−0.002 (3)
C12	0.036 (3)	0.027 (3)	0.023 (3)	−0.003 (2)	−0.001 (2)	−0.001 (2)
C13	0.026 (2)	0.014 (2)	0.035 (3)	−0.0004 (18)	0.005 (2)	0.006 (2)
C14	0.033 (3)	0.013 (2)	0.030 (3)	−0.0004 (19)	−0.004 (2)	0.005 (2)
C15	0.034 (3)	0.023 (3)	0.024 (3)	0.000 (2)	0.002 (2)	0.000 (2)
C16	0.042 (3)	0.024 (3)	0.025 (3)	0.010 (2)	0.001 (2)	−0.002 (2)
C17	0.053 (4)	0.013 (2)	0.042 (4)	0.002 (2)	−0.001 (3)	0.000 (2)
C18	0.046 (4)	0.016 (3)	0.089 (6)	−0.008 (3)	0.002 (4)	−0.008 (3)
C19	0.033 (3)	0.017 (3)	0.089 (6)	−0.005 (2)	0.006 (3)	−0.009 (3)
C20	0.027 (2)	0.016 (2)	0.024 (3)	0.0016 (18)	−0.003 (2)	−0.001 (2)
C21	0.029 (3)	0.024 (3)	0.031 (3)	−0.001 (2)	0.000 (2)	0.005 (2)
C22	0.038 (3)	0.035 (3)	0.026 (3)	0.005 (2)	−0.002 (2)	−0.003 (2)
C23	0.031 (3)	0.027 (3)	0.032 (3)	0.002 (2)	−0.007 (2)	−0.010 (2)
C24	0.036 (3)	0.019 (3)	0.041 (3)	−0.004 (2)	−0.008 (3)	−0.005 (2)
C25	0.032 (3)	0.018 (2)	0.027 (3)	−0.0022 (19)	−0.003 (2)	0.004 (2)
C26	0.032 (3)	0.023 (3)	0.027 (3)	0.001 (2)	−0.008 (2)	0.001 (2)
C27	0.050 (4)	0.021 (3)	0.030 (3)	−0.009 (2)	−0.007 (3)	0.006 (2)
C28	0.088 (6)	0.025 (3)	0.039 (4)	−0.009 (3)	−0.026 (4)	0.000 (3)
C29	0.116 (8)	0.025 (3)	0.048 (5)	0.009 (4)	−0.038 (5)	−0.011 (3)
C30	0.073 (5)	0.044 (4)	0.041 (4)	0.030 (4)	−0.015 (4)	−0.017 (3)
C31	0.045 (3)	0.034 (3)	0.032 (3)	0.013 (3)	−0.007 (3)	−0.002 (3)
C32	0.026 (3)	0.047 (4)	0.032 (3)	−0.010 (2)	−0.010 (2)	0.008 (3)
C33	0.033 (3)	0.059 (4)	0.043 (4)	0.009 (3)	−0.009 (3)	0.002 (3)
C34	0.046 (4)	0.106 (8)	0.035 (4)	−0.001 (4)	−0.001 (3)	−0.025 (5)
C35	0.062 (5)	0.108 (8)	0.027 (4)	−0.037 (5)	−0.009 (3)	0.010 (4)
C36	0.084 (6)	0.066 (5)	0.032 (4)	−0.041 (5)	−0.018 (4)	0.018 (4)
C37	0.054 (4)	0.038 (3)	0.028 (3)	−0.019 (3)	−0.013 (3)	0.006 (3)
C38	0.026 (3)	0.030 (3)	0.045 (4)	−0.004 (2)	−0.007 (3)	0.011 (3)
C39A	0.031 (4)	0.030 (4)	0.082 (6)	0.000 (3)	−0.034 (5)	0.006 (5)
C40A	0.045 (5)	0.051 (5)	0.086 (6)	−0.008 (4)	−0.019 (5)	0.012 (5)
C41A	0.049 (7)	0.043 (6)	0.115 (8)	−0.009 (5)	−0.018 (7)	−0.002 (6)
C42A	0.056 (6)	0.052 (6)	0.099 (7)	0.004 (4)	−0.046 (6)	−0.015 (6)
C43A	0.074 (7)	0.056 (6)	0.077 (6)	−0.002 (5)	−0.034 (5)	−0.003 (5)
C44A	0.054 (5)	0.039 (5)	0.077 (6)	−0.006 (4)	−0.025 (5)	−0.003 (5)
C45	0.028 (3)	0.024 (3)	0.019 (2)	0.001 (2)	0.001 (2)	−0.002 (2)
C46	0.039 (3)	0.026 (3)	0.047 (4)	0.001 (2)	0.003 (3)	0.011 (3)
C47	0.033 (3)	0.026 (3)	0.037 (3)	−0.001 (2)	−0.007 (3)	−0.006 (2)
C48	0.035 (3)	0.024 (3)	0.036 (3)	0.000 (2)	−0.008 (3)	0.001 (2)
C49	0.034 (3)	0.016 (2)	0.033 (3)	0.002 (2)	0.002 (2)	0.006 (2)
C50	0.027 (2)	0.021 (2)	0.027 (3)	0.0041 (19)	−0.001 (2)	0.005 (2)
C51	0.027 (3)	0.037 (3)	0.013 (2)	−0.002 (2)	−0.001 (2)	0.001 (2)
C52	0.026 (3)	0.043 (3)	0.038 (3)	0.003 (2)	−0.006 (3)	−0.018 (3)
C53	0.038 (3)	0.023 (3)	0.044 (4)	−0.002 (2)	−0.014 (3)	0.002 (3)

### Geometric parameters (Å, °)

**Table:**

Ru1—C46	1.872 (7)	C18—C19	1.401 (8)
Ru1—C47	1.920 (6)	C18—H18A	0.9300
Ru1—C45	1.926 (5)	C19—H19A	0.9300
Ru1—As1	2.4492 (7)	C20—C21	1.384 (7)
Ru1—Ru2	2.8598 (6)	C20—C25	1.387 (7)
Ru1—Ru3	2.8702 (6)	C21—C22	1.385 (8)
Ru2—C49	1.890 (6)	C21—H21A	0.9300
Ru2—C48	1.932 (6)	C22—C23	1.371 (8)
Ru2—C50	1.935 (5)	C22—H22A	0.9300
Ru2—As2	2.4364 (7)	C23—C24	1.393 (9)
Ru2—Ru3	2.8478 (6)	C23—H23A	0.9300
Ru3—C52	1.873 (7)	C24—C25	1.384 (7)
Ru3—C53	1.925 (6)	C24—H24A	0.9300
Ru3—C51	1.943 (6)	C25—H25A	0.9300
Ru3—P1	2.3415 (14)	C26—C31	1.393 (9)
As1—C7	1.946 (5)	C26—C27	1.397 (8)
As1—C1	1.951 (6)	C27—C28	1.385 (9)
As1—C13	1.966 (5)	C27—H27A	0.9300
As2—C20	1.944 (5)	C28—C29	1.369 (13)
As2—C14	1.948 (5)	C28—H28A	0.9300
As2—C13	1.959 (5)	C29—C30	1.359 (12)
P1—C26	1.815 (6)	C29—H29A	0.9300
P1—C32	1.835 (6)	C30—C31	1.404 (9)
P1—C38	1.861 (6)	C30—H30A	0.9300
S1—C39B	1.55 (7)	C31—H31A	0.9300
S1—C39A	1.775 (11)	C32—C33	1.359 (10)
S1—C38	1.822 (6)	C32—C37	1.405 (9)
O1—C45	1.150 (6)	C33—C34	1.402 (10)
O2—C46	1.152 (8)	C33—H33A	0.9300
O3—C47	1.153 (7)	C34—C35	1.373 (13)
O4—C48	1.143 (7)	C34—H34A	0.9300
O5—C49	1.143 (7)	C35—C36	1.370 (13)
O6—C50	1.144 (6)	C35—H35A	0.9300
O7—C51	1.141 (7)	C36—C37	1.398 (9)
O8—C52	1.159 (8)	C36—H36A	0.9300
O9—C53	1.148 (7)	C37—H37A	0.9300
C1—C2	1.369 (8)	C38—H38A	0.9700
C1—C6	1.379 (9)	C38—H38B	0.9700
C2—C3	1.396 (10)	C39A—C44A	1.372 (18)
C2—H2A	0.9300	C39A—C40A	1.374 (15)
C3—C4	1.359 (11)	C40A—C41A	1.378 (18)
C3—H3A	0.9300	C40A—H40A	0.9300
C4—C5	1.381 (10)	C41A—C42A	1.32 (2)
C4—H4A	0.9300	C41A—H41A	0.9300
C5—C6	1.379 (9)	C42A—C43A	1.386 (18)
C5—H5A	0.9300	C42A—H42A	0.9300
C6—H6A	0.9300	C43A—C44A	1.398 (14)
C7—C12	1.386 (7)	C43A—H43A	0.9300
C7—C8	1.393 (8)	C44A—H44A	0.9300
C8—C9	1.388 (9)	C39B—C40B	1.30 (7)
C8—H8A	0.9300	C39B—C44B	1.38 (7)
C9—C10	1.379 (9)	C40B—C41B	1.42 (6)
C9—H9A	0.9300	C40B—H40B	0.9300
C10—C11	1.374 (9)	C41B—C42B	1.11 (7)
C10—H10A	0.9300	C41B—H41B	0.9300
C11—C12	1.391 (8)	C42B—C43B	1.28 (7)
C11—H11A	0.9300	C42B—H42B	0.9300
C12—H12A	0.9300	C43B—C44B	1.46 (8)
C13—H13A	0.9700	C43B—H43B	0.9300
C13—H13B	0.9700	C44B—H44B	0.9300
C14—C19	1.373 (8)	Cl1A—C54A	1.755 (16)
C14—C15	1.381 (8)	Cl2A—C54A	1.744 (16)
C15—C16	1.395 (7)	Cl3A—C54A	1.681 (17)
C15—H15A	0.9300	C54A—H54A	0.9800
C16—C17	1.366 (8)	Cl1B—C54B	1.756 (18)
C16—H16A	0.9300	Cl2B—C54B	1.761 (18)
C17—C18	1.354 (10)	Cl3B—C54B	1.753 (19)
C17—H17A	0.9300	C54B—H54B	0.9800
			
C46—Ru1—C47	94.4 (3)	C19—C18—H18A	119.9
C46—Ru1—C45	89.7 (2)	C14—C19—C18	120.1 (6)
C47—Ru1—C45	170.9 (2)	C14—C19—H19A	119.9
C46—Ru1—As1	99.1 (2)	C18—C19—H19A	119.9
C47—Ru1—As1	86.75 (18)	C21—C20—C25	119.8 (5)
C45—Ru1—As1	100.64 (15)	C21—C20—As2	120.8 (4)
C46—Ru1—Ru2	167.7 (2)	C25—C20—As2	119.3 (4)
C47—Ru1—Ru2	78.55 (18)	C20—C21—C22	119.9 (5)
C45—Ru1—Ru2	95.89 (16)	C20—C21—H21A	120.0
As1—Ru1—Ru2	90.56 (2)	C22—C21—H21A	120.0
C46—Ru1—Ru3	111.1 (2)	C23—C22—C21	120.4 (6)
C47—Ru1—Ru3	91.73 (19)	C23—C22—H22A	119.8
C45—Ru1—Ru3	79.18 (16)	C21—C22—H22A	119.8
As1—Ru1—Ru3	149.72 (2)	C22—C23—C24	120.0 (5)
Ru2—Ru1—Ru3	59.602 (15)	C22—C23—H23A	120.0
C49—Ru2—C48	89.1 (3)	C24—C23—H23A	120.0
C49—Ru2—C50	93.1 (2)	C25—C24—C23	119.7 (5)
C48—Ru2—C50	174.3 (2)	C25—C24—H24A	120.1
C49—Ru2—As2	100.44 (16)	C23—C24—H24A	120.1
C48—Ru2—As2	91.48 (18)	C24—C25—C20	120.0 (5)
C50—Ru2—As2	93.30 (16)	C24—C25—H25A	120.0
C49—Ru2—Ru3	105.65 (17)	C20—C25—H25A	120.0
C48—Ru2—Ru3	81.14 (17)	C31—C26—C27	119.7 (6)
C50—Ru2—Ru3	93.17 (15)	C31—C26—P1	121.7 (5)
As2—Ru2—Ru3	152.70 (2)	C27—C26—P1	118.5 (5)
C49—Ru2—Ru1	162.64 (16)	C28—C27—C26	119.8 (7)
C48—Ru2—Ru1	98.13 (18)	C28—C27—H27A	120.1
C50—Ru2—Ru1	78.30 (16)	C26—C27—H27A	120.1
As2—Ru2—Ru1	95.16 (2)	C29—C28—C27	120.7 (7)
Ru3—Ru2—Ru1	60.382 (15)	C29—C28—H28A	119.7
C52—Ru3—C53	91.4 (3)	C27—C28—H28A	119.7
C52—Ru3—C51	89.8 (2)	C30—C29—C28	120.0 (7)
C53—Ru3—C51	174.6 (2)	C30—C29—H29A	120.0
C52—Ru3—P1	99.78 (19)	C28—C29—H29A	120.0
C53—Ru3—P1	90.13 (17)	C29—C30—C31	121.4 (8)
C51—Ru3—P1	94.84 (16)	C29—C30—H30A	119.3
C52—Ru3—Ru2	101.10 (19)	C31—C30—H30A	119.3
C53—Ru3—Ru2	94.15 (17)	C26—C31—C30	118.4 (7)
C51—Ru3—Ru2	80.47 (15)	C26—C31—H31A	120.8
P1—Ru3—Ru2	158.56 (4)	C30—C31—H31A	120.8
C52—Ru3—Ru1	159.0 (2)	C33—C32—C37	118.8 (6)
C53—Ru3—Ru1	81.7 (2)	C33—C32—P1	118.8 (5)
C51—Ru3—Ru1	95.31 (15)	C37—C32—P1	122.3 (5)
P1—Ru3—Ru1	100.01 (4)	C32—C33—C34	121.7 (7)
Ru2—Ru3—Ru1	60.017 (15)	C32—C33—H33A	119.1
C7—As1—C1	102.1 (2)	C34—C33—H33A	119.1
C7—As1—C13	102.7 (2)	C35—C34—C33	119.4 (8)
C1—As1—C13	101.3 (2)	C35—C34—H34A	120.3
C7—As1—Ru1	123.75 (16)	C33—C34—H34A	120.3
C1—As1—Ru1	114.40 (17)	C36—C35—C34	119.9 (7)
C13—As1—Ru1	109.86 (17)	C36—C35—H35A	120.0
C20—As2—C14	101.4 (2)	C34—C35—H35A	120.0
C20—As2—C13	101.2 (2)	C35—C36—C37	120.9 (8)
C14—As2—C13	103.6 (2)	C35—C36—H36A	119.5
C20—As2—Ru2	120.55 (15)	C37—C36—H36A	119.5
C14—As2—Ru2	114.75 (17)	C36—C37—C32	119.3 (7)
C13—As2—Ru2	113.13 (15)	C36—C37—H37A	120.4
C26—P1—C32	103.8 (3)	C32—C37—H37A	120.4
C26—P1—C38	105.0 (3)	S1—C38—P1	114.7 (3)
C32—P1—C38	99.7 (3)	S1—C38—H38A	108.6
C26—P1—Ru3	114.58 (17)	P1—C38—H38A	108.6
C32—P1—Ru3	118.0 (2)	S1—C38—H38B	108.6
C38—P1—Ru3	113.85 (18)	P1—C38—H38B	108.6
C39B—S1—C39A	22 (2)	H38A—C38—H38B	107.6
C39B—S1—C38	103 (2)	C44A—C39A—C40A	118.6 (11)
C39A—S1—C38	103.6 (4)	C44A—C39A—S1	118.1 (8)
C2—C1—C6	119.1 (6)	C40A—C39A—S1	123.3 (11)
C2—C1—As1	118.5 (5)	C39A—C40A—C41A	120.3 (14)
C6—C1—As1	122.2 (4)	C39A—C40A—H40A	119.9
C1—C2—C3	120.3 (7)	C41A—C40A—H40A	119.9
C1—C2—H2A	119.8	C42A—C41A—C40A	121.7 (14)
C3—C2—H2A	119.8	C42A—C41A—H41A	119.1
C4—C3—C2	120.7 (7)	C40A—C41A—H41A	119.1
C4—C3—H3A	119.6	C41A—C42A—C43A	119.8 (12)
C2—C3—H3A	119.6	C41A—C42A—H42A	120.1
C3—C4—C5	118.7 (7)	C43A—C42A—H42A	120.1
C3—C4—H4A	120.6	C42A—C43A—C44A	119.1 (13)
C5—C4—H4A	120.6	C42A—C43A—H43A	120.4
C6—C5—C4	121.0 (7)	C44A—C43A—H43A	120.4
C6—C5—H5A	119.5	C39A—C44A—C43A	120.4 (11)
C4—C5—H5A	119.5	C39A—C44A—H44A	119.8
C5—C6—C1	120.1 (6)	C43A—C44A—H44A	119.8
C5—C6—H6A	120.0	C40B—C39B—C44B	112 (6)
C1—C6—H6A	120.0	C40B—C39B—S1	123 (5)
C12—C7—C8	119.4 (5)	C44B—C39B—S1	124 (5)
C12—C7—As1	119.7 (4)	C39B—C40B—C41B	123 (5)
C8—C7—As1	120.9 (4)	C39B—C40B—H40B	118.3
C9—C8—C7	119.6 (6)	C41B—C40B—H40B	118.3
C9—C8—H8A	120.2	C42B—C41B—C40B	122 (6)
C7—C8—H8A	120.2	C42B—C41B—H41B	119.0
C10—C9—C8	120.6 (6)	C40B—C41B—H41B	119.0
C10—C9—H9A	119.7	C41B—C42B—C43B	123 (7)
C8—C9—H9A	119.7	C41B—C42B—H42B	118.5
C11—C10—C9	120.0 (6)	C43B—C42B—H42B	118.5
C11—C10—H10A	120.0	C42B—C43B—C44B	117 (6)
C9—C10—H10A	120.0	C42B—C43B—H43B	121.3
C10—C11—C12	120.0 (6)	C44B—C43B—H43B	121.3
C10—C11—H11A	120.0	C39B—C44B—C43B	119 (5)
C12—C11—H11A	120.0	C39B—C44B—H44B	120.3
C7—C12—C11	120.4 (5)	C43B—C44B—H44B	120.3
C7—C12—H12A	119.8	O1—C45—Ru1	173.9 (5)
C11—C12—H12A	119.8	O2—C46—Ru1	178.0 (6)
As2—C13—As1	109.2 (2)	O3—C47—Ru1	172.2 (6)
As2—C13—H13A	109.8	O4—C48—Ru2	173.1 (5)
As1—C13—H13A	109.8	O5—C49—Ru2	179.3 (6)
As2—C13—H13B	109.8	O6—C50—Ru2	174.9 (5)
As1—C13—H13B	109.8	O7—C51—Ru3	174.5 (5)
H13A—C13—H13B	108.3	O8—C52—Ru3	178.6 (6)
C19—C14—C15	119.4 (5)	O9—C53—Ru3	173.8 (6)
C19—C14—As2	121.5 (4)	Cl3A—C54A—Cl2A	97.4 (11)
C15—C14—As2	118.8 (4)	Cl3A—C54A—Cl1A	112.0 (14)
C14—C15—C16	119.6 (5)	Cl2A—C54A—Cl1A	116.4 (14)
C14—C15—H15A	120.2	Cl3A—C54A—H54A	110.1
C16—C15—H15A	120.2	Cl2A—C54A—H54A	110.1
C17—C16—C15	120.6 (6)	Cl1A—C54A—H54A	110.1
C17—C16—H16A	119.7	Cl3B—C54B—Cl1B	114.8 (17)
C15—C16—H16A	119.7	Cl3B—C54B—Cl2B	102.4 (15)
C18—C17—C16	120.1 (5)	Cl1B—C54B—Cl2B	110.5 (17)
C18—C17—H17A	119.9	Cl3B—C54B—H54B	109.6
C16—C17—H17A	119.9	Cl1B—C54B—H54B	109.6
C17—C18—C19	120.2 (6)	Cl2B—C54B—H54B	109.6
C17—C18—H18A	119.9		
			
C46—Ru1—Ru2—C49	−82.0 (11)	C13—As1—C1—C2	64.8 (6)
C47—Ru1—Ru2—C49	−137.8 (6)	Ru1—As1—C1—C2	−53.3 (6)
C45—Ru1—Ru2—C49	34.9 (6)	C7—As1—C1—C6	−13.9 (5)
As1—Ru1—Ru2—C49	135.7 (6)	C13—As1—C1—C6	−119.7 (5)
Ru3—Ru1—Ru2—C49	−38.9 (6)	Ru1—As1—C1—C6	122.2 (5)
C46—Ru1—Ru2—C48	31.8 (9)	C6—C1—C2—C3	0.5 (11)
C47—Ru1—Ru2—C48	−24.0 (3)	As1—C1—C2—C3	176.2 (6)
C45—Ru1—Ru2—C48	148.7 (2)	C1—C2—C3—C4	−1.1 (13)
As1—Ru1—Ru2—C48	−110.55 (18)	C2—C3—C4—C5	0.8 (12)
Ru3—Ru1—Ru2—C48	74.92 (18)	C3—C4—C5—C6	0.1 (11)
C46—Ru1—Ru2—C50	−143.6 (9)	C4—C5—C6—C1	−0.7 (10)
C47—Ru1—Ru2—C50	160.6 (2)	C2—C1—C6—C5	0.4 (10)
C45—Ru1—Ru2—C50	−26.7 (2)	As1—C1—C6—C5	−175.1 (5)
As1—Ru1—Ru2—C50	74.01 (16)	C1—As1—C7—C12	107.6 (5)
Ru3—Ru1—Ru2—C50	−100.53 (16)	C13—As1—C7—C12	−147.7 (5)
C46—Ru1—Ru2—As2	124.0 (9)	Ru1—As1—C7—C12	−23.0 (5)
C47—Ru1—Ru2—As2	68.27 (19)	C1—As1—C7—C8	−72.4 (5)
C45—Ru1—Ru2—As2	−119.06 (15)	C13—As1—C7—C8	32.3 (5)
As1—Ru1—Ru2—As2	−18.31 (2)	Ru1—As1—C7—C8	157.0 (4)
Ru3—Ru1—Ru2—As2	167.16 (2)	C12—C7—C8—C9	0.2 (9)
C46—Ru1—Ru2—Ru3	−43.1 (9)	As1—C7—C8—C9	−179.9 (5)
C47—Ru1—Ru2—Ru3	−98.89 (19)	C7—C8—C9—C10	1.9 (10)
C45—Ru1—Ru2—Ru3	73.78 (15)	C8—C9—C10—C11	−2.8 (10)
As1—Ru1—Ru2—Ru3	174.54 (2)	C9—C10—C11—C12	1.6 (10)
C49—Ru2—Ru3—C52	−21.0 (3)	C8—C7—C12—C11	−1.4 (9)
C48—Ru2—Ru3—C52	65.6 (3)	As1—C7—C12—C11	178.7 (5)
C50—Ru2—Ru3—C52	−115.1 (3)	C10—C11—C12—C7	0.5 (9)
As2—Ru2—Ru3—C52	141.4 (2)	C20—As2—C13—As1	−95.6 (3)
Ru1—Ru2—Ru3—C52	170.2 (2)	C14—As2—C13—As1	159.7 (3)
C49—Ru2—Ru3—C53	−113.2 (3)	Ru2—As2—C13—As1	34.8 (3)
C48—Ru2—Ru3—C53	−26.7 (3)	C7—As1—C13—As2	81.0 (3)
C50—Ru2—Ru3—C53	152.6 (3)	C1—As1—C13—As2	−173.7 (3)
As2—Ru2—Ru3—C53	49.1 (2)	Ru1—As1—C13—As2	−52.4 (3)
Ru1—Ru2—Ru3—C53	78.0 (2)	C20—As2—C14—C19	−145.9 (6)
C49—Ru2—Ru3—C51	66.9 (2)	C13—As2—C14—C19	−41.3 (6)
C48—Ru2—Ru3—C51	153.5 (3)	Ru2—As2—C14—C19	82.6 (6)
C50—Ru2—Ru3—C51	−27.2 (2)	C20—As2—C14—C15	40.4 (5)
As2—Ru2—Ru3—C51	−130.70 (17)	C13—As2—C14—C15	145.0 (5)
Ru1—Ru2—Ru3—C51	−101.85 (16)	Ru2—As2—C14—C15	−91.1 (4)
C49—Ru2—Ru3—P1	145.8 (2)	C19—C14—C15—C16	−0.7 (9)
C48—Ru2—Ru3—P1	−127.7 (2)	As2—C14—C15—C16	173.1 (4)
C50—Ru2—Ru3—P1	51.6 (2)	C14—C15—C16—C17	0.4 (9)
As2—Ru2—Ru3—P1	−51.88 (12)	C15—C16—C17—C18	0.5 (10)
Ru1—Ru2—Ru3—P1	−23.03 (10)	C16—C17—C18—C19	−1.1 (12)
C49—Ru2—Ru3—Ru1	168.78 (17)	C15—C14—C19—C18	0.2 (11)
C48—Ru2—Ru3—Ru1	−104.7 (2)	As2—C14—C19—C18	−173.5 (6)
C50—Ru2—Ru3—Ru1	74.62 (17)	C17—C18—C19—C14	0.8 (12)
As2—Ru2—Ru3—Ru1	−28.85 (5)	C14—As2—C20—C21	56.9 (5)
C46—Ru1—Ru3—C52	143.3 (5)	C13—As2—C20—C21	−49.6 (5)
C47—Ru1—Ru3—C52	47.9 (5)	Ru2—As2—C20—C21	−175.1 (4)
C45—Ru1—Ru3—C52	−131.2 (5)	C14—As2—C20—C25	−124.5 (4)
As1—Ru1—Ru3—C52	−38.6 (5)	C13—As2—C20—C25	129.0 (4)
Ru2—Ru1—Ru3—C52	−27.8 (5)	Ru2—As2—C20—C25	3.4 (5)
C46—Ru1—Ru3—C53	71.4 (3)	C25—C20—C21—C22	−1.6 (8)
C47—Ru1—Ru3—C53	−24.0 (2)	As2—C20—C21—C22	176.9 (4)
C45—Ru1—Ru3—C53	156.8 (2)	C20—C21—C22—C23	−0.8 (9)
As1—Ru1—Ru3—C53	−110.57 (18)	C21—C22—C23—C24	2.5 (9)
Ru2—Ru1—Ru3—C53	−99.69 (17)	C22—C23—C24—C25	−1.7 (9)
C46—Ru1—Ru3—C51	−113.2 (3)	C23—C24—C25—C20	−0.7 (9)
C47—Ru1—Ru3—C51	151.4 (2)	C21—C20—C25—C24	2.4 (8)
C45—Ru1—Ru3—C51	−27.7 (2)	As2—C20—C25—C24	−176.2 (4)
As1—Ru1—Ru3—C51	64.89 (17)	C32—P1—C26—C31	−122.1 (5)
Ru2—Ru1—Ru3—C51	75.77 (16)	C38—P1—C26—C31	−17.8 (5)
C46—Ru1—Ru3—P1	−17.3 (2)	Ru3—P1—C26—C31	107.8 (4)
C47—Ru1—Ru3—P1	−112.71 (17)	C32—P1—C26—C27	61.4 (5)
C45—Ru1—Ru3—P1	68.16 (16)	C38—P1—C26—C27	165.6 (4)
As1—Ru1—Ru3—P1	160.77 (5)	Ru3—P1—C26—C27	−68.7 (5)
Ru2—Ru1—Ru3—P1	171.65 (4)	C31—C26—C27—C28	1.0 (8)
C46—Ru1—Ru3—Ru2	171.0 (2)	P1—C26—C27—C28	177.6 (5)
C47—Ru1—Ru3—Ru2	75.64 (17)	C26—C27—C28—C29	0.2 (9)
C45—Ru1—Ru3—Ru2	−103.49 (16)	C27—C28—C29—C30	−0.4 (11)
As1—Ru1—Ru3—Ru2	−10.89 (4)	C28—C29—C30—C31	−0.7 (11)
C46—Ru1—As1—C7	107.2 (3)	C27—C26—C31—C30	−2.0 (8)
C47—Ru1—As1—C7	−158.9 (3)	P1—C26—C31—C30	−178.5 (5)
C45—Ru1—As1—C7	15.7 (3)	C29—C30—C31—C26	1.9 (10)
Ru2—Ru1—As1—C7	−80.36 (19)	C26—P1—C32—C33	−173.6 (5)
Ru3—Ru1—As1—C7	−71.0 (2)	C38—P1—C32—C33	78.1 (5)
C46—Ru1—As1—C1	−18.2 (3)	Ru3—P1—C32—C33	−45.6 (5)
C47—Ru1—As1—C1	75.7 (3)	C26—P1—C32—C37	10.4 (6)
C45—Ru1—As1—C1	−109.7 (2)	C38—P1—C32—C37	−97.9 (5)
Ru2—Ru1—As1—C1	154.23 (19)	Ru3—P1—C32—C37	138.4 (5)
Ru3—Ru1—As1—C1	163.60 (19)	C37—C32—C33—C34	−0.1 (10)
C46—Ru1—As1—C13	−131.3 (2)	P1—C32—C33—C34	−176.2 (6)
C47—Ru1—As1—C13	−37.4 (2)	C32—C33—C34—C35	−1.1 (11)
C45—Ru1—As1—C13	137.2 (2)	C33—C34—C35—C36	0.9 (12)
Ru2—Ru1—As1—C13	41.14 (16)	C34—C35—C36—C37	0.6 (12)
Ru3—Ru1—As1—C13	50.51 (17)	C35—C36—C37—C32	−1.8 (11)
C49—Ru2—As2—C20	−58.0 (2)	C33—C32—C37—C36	1.5 (10)
C48—Ru2—As2—C20	−147.4 (3)	P1—C32—C37—C36	177.5 (5)
C50—Ru2—As2—C20	35.8 (2)	C39B—S1—C38—P1	−142 (2)
Ru3—Ru2—As2—C20	139.24 (18)	C39A—S1—C38—P1	−119.5 (5)
Ru1—Ru2—As2—C20	114.33 (18)	C26—P1—C38—S1	−49.4 (4)
C49—Ru2—As2—C14	63.6 (2)	C32—P1—C38—S1	57.8 (4)
C48—Ru2—As2—C14	−25.8 (3)	Ru3—P1—C38—S1	−175.6 (3)
C50—Ru2—As2—C14	157.4 (2)	C39B—S1—C39A—C44A	−172 (7)
Ru3—Ru2—As2—C14	−99.15 (19)	C38—S1—C39A—C44A	97.0 (8)
Ru1—Ru2—As2—C14	−124.06 (18)	C39B—S1—C39A—C40A	6(7)
C49—Ru2—As2—C13	−177.8 (2)	C38—S1—C39A—C40A	−85.4 (9)
C48—Ru2—As2—C13	92.8 (3)	C44A—C39A—C40A—C41A	0.9 (16)
C50—Ru2—As2—C13	−84.0 (2)	S1—C39A—C40A—C41A	−176.7 (9)
Ru3—Ru2—As2—C13	19.45 (18)	C39A—C40A—C41A—C42A	1.5 (18)
Ru1—Ru2—As2—C13	−5.47 (17)	C40A—C41A—C42A—C43A	−2.8 (18)
C52—Ru3—P1—C26	122.3 (3)	C41A—C42A—C43A—C44A	1.8 (17)
C53—Ru3—P1—C26	−146.3 (3)	C40A—C39A—C44A—C43A	−1.8 (15)
C51—Ru3—P1—C26	31.6 (3)	S1—C39A—C44A—C43A	175.9 (8)
Ru2—Ru3—P1—C26	−44.5 (3)	C42A—C43A—C44A—C39A	0.5 (16)
Ru1—Ru3—P1—C26	−64.7 (2)	C39A—S1—C39B—C40B	168 (12)
C52—Ru3—P1—C32	−0.5 (3)	C38—S1—C39B—C40B	−98 (6)
C53—Ru3—P1—C32	91.0 (3)	C39A—S1—C39B—C44B	0(3)
C51—Ru3—P1—C32	−91.1 (3)	C38—S1—C39B—C44B	94 (6)
Ru2—Ru3—P1—C32	−167.3 (2)	C44B—C39B—C40B—C41B	−7(8)
Ru1—Ru3—P1—C32	172.6 (2)	S1—C39B—C40B—C41B	−176 (4)
C52—Ru3—P1—C38	−116.8 (3)	C39B—C40B—C41B—C42B	−5(9)
C53—Ru3—P1—C38	−25.4 (3)	C40B—C41B—C42B—C43B	18 (11)
C51—Ru3—P1—C38	152.5 (3)	C41B—C42B—C43B—C44B	−18 (11)
Ru2—Ru3—P1—C38	76.4 (3)	C40B—C39B—C44B—C43B	6(8)
Ru1—Ru3—P1—C38	56.3 (2)	S1—C39B—C44B—C43B	175 (5)
C7—As1—C1—C2	170.6 (6)	C42B—C43B—C44B—C39B	5(9)

### Hydrogen-bond geometry (Å, °)

**Table:**

Cg1, Cg2 and Cg3 are the centroids of the C39A–C44A, C14–C19 and C32–C37benzene rings, respectively.

**Table:**

*D*—H···*A*	*D*—H	H···*A*	*D*···*A*	*D*—H···*A*
C18—H18A···O1^i^	0.93	2.53	3.457 (7)	175
C29—H29A···O3^ii^	0.93	2.45	3.331 (9)	158
C3—H3A···Cg1^iii^	0.93	2.82	3.654 (9)	151
C23—H23A···Cg2^iv^	0.93	2.87	3.691 (7)	147
C44A—H44A···Cg3	0.93	2.76	3.526 (12)	141

Symmetry codes: (i) *x*, *y*+1, *z*; (ii) *x*, *y*−1, *z*; (iii) *x*, −*y*−1/2, *z*−3/2; (iv) −*x*+1, *y*−1/2, −*z*+1/2.
